# Repeated Ethanol Exposure Alters DNA Methylation Status and Dynorphin/Kappa-Opioid Receptor Expression in Nucleus Accumbens of Alcohol-Preferring AA Rats

**DOI:** 10.3389/fgene.2021.750142

**Published:** 2021-11-24

**Authors:** Kerly Niinep, Kaili Anier, Tony Eteläinen, Petteri Piepponen, Anti Kalda

**Affiliations:** ^1^ Department of Pharmacology, University of Tartu, Tartu, Estonia; ^2^ Department of Pharmacology and Pharmacotherapy, University of Helsinki, Helsinki, Finland

**Keywords:** ethanol, epigenetics, nucleus accumbens, kappa opioid receptor, dynorphin, DNA methyltransferase, DNA demethylase, rats

## Abstract

Growing evidence suggests that epigenetic mechanisms, such as DNA methylation and demethylation, and histone modifications, are involved in the development of alcohol and drug addiction. However, studies of alcohol use disorder (AUD) that are focused on epigenetic DNA modifications and gene expression changes remain conflicting. Our aim was to study the effect of repeated ethanol consumption on epigenetic regulatory enzymes such as DNA methyltransferase and demethylase enzymes and whether those changes affected dynorphin/kappa-opioid receptor system in the Nucleus Accumbens (NAc). Two groups of male alcohol-preferring Alko Alcohol (AA) rats, rats which are selectively bred for high voluntary alcohol consumption and one group of male Wistar rats were used. The first group of AA rats had access to alcohol (10% ethanol solution) for 90 min on Mondays, Wednesdays and Fridays over a period of 3 weeks to establish a stable baseline of ethanol intake (AA-ethanol). The second group of AA rats (AA-water) and the Wistar rats (Wistar-water) were provided with water. Using qPCR, we found that voluntary alcohol drinking increased *Dnmt1*, *−3a*, and *−3b* mRNA levels and did not affect *Tet* family transcripts in the AA-ethanol group when compared with AA- and Wistar-water rats. DNMT and TET enzymatic activity measurements showed similar results to qPCR, where DNMT activity was increased in AA-ethanol group compared with AA-water and Wistar-water groups, with no statistically significant difference between groups in TET enzyme activity. In line with previous data, we found an increased percentage of global DNA methylation and hydroxymethylation in the AA-ethanol group compared with control rats. Finally, we investigated changes of selected candidate genes from dynorphin/kappa-opioid receptor system (*Pdyn, Kor*) and *Dnmt3a* genes that might be important in AUD-related behaviour. Our gene expression and promoter methylation analysis revealed a significant increase in the mRNA levels of *Pdyn, Kor,* and *Dnmt3a* in the AA-ethanol group, however, these changes can only be partially associate with the aberrant DNA methylation in promoter areas of the selected candidate genes. Thus, our findings suggest that the aberrant DNA methylation is rather one of the several mechanisms involved in gene expression regulation in AA rat model.

## Introduction

Alcohol use disorder (AUD) is complex psychiatric disorder that is characterized by excessive alcohol drinking, alcohol dependence and relapses even after long periods of abstinence (Siomek-Gorecka et al., 2021). It is a devastating public health problem and according to a WHO Global status report on alcohol and health in 2018, harmful use of alcohol resulted in about three million deaths worldwide in 2016 (World Health Organization, 2018). Alcohol and other drugs of abuse have been shown to change the synaptic plasticity and function of neurons in specific brain regions, resulting in long term changes at molecular, cellular and behavioural level (Berkel and Pandey, 2017). It has been shown that during the development of AUD, both genetic and environmental factors play an important role (Siomek-Gorecka et al., 2021). The fact that alcoholism tends to run in families has long been known and almost all twin studies have shown that heritability in AUD ranges from 40 to 60% (Ducci and Goldman, 2008; Verhulst et al., 2015).

In this study, we focused on alcohol-induced aberrant DNA methylation. DNA methylation is considered to be the most stable epigenetic modification with critical roles in brain development (Lister et al., 2013; Kinde et al., 2015). DNA methylation has been an important area of research also in the study of molecular mechanism to psychiatric disorders (Liu et al., 2018). Accumulated evidence has suggested that abnormalities in global methylation and methylation of genes could play a role in the pathogenesis of many forms of mental illness (Kuehner et al., 2019). In mammals, DNA methylation occurs predominantly at cytosine-guanine (CpG) dinucleotide sites, of which approximately 60–80% are methylated (Wu and Zhang, 2017). Furthermore, these CpG-rich regions are often methylated near gene promoters, which typically causes gene silencing (Deaton and Bird, 2011; Li and Zhang, 2014). DNA methylation is catalysed by DNA methyltransferases (DNMTs), including DNMT1 (maintenance DNMT), DNMT3a and DNMT3b (*de novo* DNMT) (Okano et al., 1999; Bestor, 2000; Goll and Bestor, 2005). To establish DNA methylation, DNMTs relocate the methyl group taken from S-adenosylmethionine (SAM) and covalently attach it to the fifth carbon of the pyrimidine ring of cytosine, forming 5-methylcytosine (5mC) (Jaenisch and Bird, 2003; Moore et al., 2012).

DNA demethylation caused by removal of the 5mC modification, can be performed in several ways. First, the modification can be removed by passive demethylation, where methylation maintenance process is impaired and no new 5mC marks are copied during DNA replication. Second, ten-eleven translocation enzymes (TET1–3) actively demethylate DNA. TET enzymes can mediate the oxidation of 5mC in order to generate hydroxymethylcytosine (5hmC) that can be further modified (Wu and Zhang, 2017). Previously the 5hmC was considered only as a transient marker of demethylation, but now it is treated as a stable epigenetic mark itself (Hahn et al., 2014). Several studies have shown that 5hmC accounts for approximately 40% of epigenetic cytosine modifications in the brain, which is a much higher density than in any other tissue found in the body (Li and Liu, 2011; Loh et al., 2017). 5hmC is found to be highly enriched within active gene promoters and in gene bodies of highly expressed genes (Wu et al., 2011). Several studies indicate that both hypomethylation and hypermethylation can be observed in postmortem human alcoholic brain (Manzardo et al., 2012; Wang et al., 2016). Although previous reports suggest a role of DNA methylation in alcohol-related behaviours, the exact molecular mechanisms by which DNA methylation contributes to long-term neuroadaptations in alcohol dependence are far more complex and remain to be clarified (Tulisiak et al., 2017; Pucci et al., 2019).

Alcohol affects many parts of the brain and exerts its effects through different neurotransmitter systems. One of the systems affected by ethanol is the endogenous opioid system (EOS), that has been shown to play critical role in development of alcohol and other drug addictions (Gianoulakis, 2001; Sirohi et al., 2012). In alcohol addiction, ethanol consumption abnormally stimulates the release of endogenous opioid peptides and activates their main receptors in the brain (Le Merrer et al., 2009; Erikson et al., 2018). The three main endogenous ligands and their receptors are the beta-endorphins (βEND), which binds to mu (*µ*) opioid receptor (MOR), the enkephalins (ENK), which binds to delta (*δ*) opioid receptors (DOR) and the dynorphins (DYN), which binds to kappa (*κ*) opioid receptor (KOR) (Sirohi et al., 2012). These endogenous ligands (βEND, ENK, and DYN) are produced from larger precursor peptides known as proopiomelanocortin (POMC), preproenkephalin (PENK) and preprodynorphin (PDYN) by proteolytic cleavage.

The drugs of abuse, including alcohol, induce an increase in the extracellular concentration of dopamine in the Nucleus Accumbens (NAc), and it was suggested that may mediate their rewarding and reinforcing properties (Di Chiara and Imperato, 1985, 1988; Le Merrer et al., 2009). Additionally, activation of MOR and DOR in the NAc enhances the extracellular concentration of dopamine in the NAc (Latimer et al., 1987; Di Chiara and Imperato, 1988; Longoni et al., 1991). Given the role of NAc in different affective and addiction disorders, the presence of KOR/DYN system in the NAc and the changed regulation of KOR/DYN in the brain during AUD, it is feasible that KOR/DYN changes in the brain are altered by epigenetic modification and neuroadaptation caused by long term alcohol use (Wee and Koob, 2010). Therefore, our aim for this study was to replicate and extend previous findings on the aberrant DNA methylation and expression of selected genes in the NAc, a critical region of the reward pathway, following intermittent voluntary ethanol intake. We hypothesized that long-term gene expression changes in the brain, *via* epigenetic modifications, underlie molecular mechanisms of AUD and relapses after abstinence. To test this hypothesis, we used two different rat strains, the standard laboratory Wistar rat and the alcohol preferring Alko Alcohol (AA) rat. The AA rat line is produced by selective breeding based on high voluntary alcohol consumption, and therefore, these rats are naturally prone to high alcohol consumption, which is hypothesized to occur due to abnormal function of opioidergic mechanisms (Eriksson, 1968; Hyytiä and Sinclair, 1989; Sommer et al., 2006; Oinio et al., 2021). Alterations in *Kor/Pdyn* and *Dnmt3a* mRNA, promoter methylation and hydroxymethylation levels were determined. Furthermore, we analysed global DNA methylation and hydroxymethylation, DNMT/TET activity and mRNA levels of other AUD-relevant genes from EOS and dopamine systems.

## Materials and Methods

### Animals

30 male alcohol-preferring (Alko, Alcohol; AA) rats (University of Helsinki, Finland) from generations F124.1 and 10 regular Wistar male rats (Harlan Laboratories Ltd., Netherlands) were used in the study. Before ethanol intake treatment AA rats were separated into two groups, first AA-ethanol group (Alko, Alcohol rats in ethanol treatment group) and second AA-water (Alko, Alcohol rats in water treatment group). The rats were 3 months old and weighed at least 250 g at the onset of the experiments. The rats were housed in standard individually ventilated cages in groups of 2–3 rats per cage and the experiments were performed in reversed 12/12 h light/dark cycle (lights off at 8 am). Temperature was maintained at 22 ± 1°C. Water and rat chow were available *ad libitum*. Animal experiments were conducted according to the 3R principles of the EU directive 2010/63/EU governing the care and use of experimental animals and following local laws and regulations [Finnish Act on the Protection of Animals Used for Scientific or Educational Purposes (497/2013), Government Decree on the Protection of Animals Used for Scientific or Educational Purposes (564/2013)]. The protocols were authorized by the national Animal Experiment Board of Finland (license: ESAVI/10030/2018).

### Ethanol Intake

The AA-ethanol group rats were trained to drink 10% ethanol solution using an intermittent, time-restricted two-bottle choice ethanol access paradigm [modified from (Uhari-Väänänen et al., 2018)]. To monitor ethanol intake, rats were transferred individually to wire mesh cages (38 × 21 × 19 cm) within 1 h after the lights were turned off. Ethanol was available for 90 min three times a week on Mondays, Wednesdays, and Fridays for 3 weeks. The position of the ethanol- and water-containing bottles were altered on each session to prevent side preference.

### Tissue Isolation

The brains were harvested and snap frozen in isopentane chilled with dry ice. Immediately after freezing, the brains were stored at −80°C until RNA, DNA or nuclear extracts were prepared. The NAc was dissected on ice using a rounded puncher (inner diameter of 1.5 mm) with a rat brain atlas as a guide (Paxinos and Watson, 2007).

### RNA Isolation and qPCR

For RNA isolation, *RNeasy Mini Kit* (QIAGEN, Hilden, Germany) was used according to the manufacturer’s instructions. For the generation of cDNA, 0.2 μg RNA and RevertAid First Strand cDNA Synthesis Kit (Thermo Scientific, Waltham, MA, United States) was used according to the manufacturer’s instructions. qPCR was performed on a QuantStudio 12K Flex Real-Time PCR system (Thermo Fisher Scientific, Waltham, MA, United States) using *SYBR Green RT-PCR Master Mix* (Thermo Fisher Scientific, Waltham, MA, United States) and previously designed primers (Table 1 and Supplementary Table S1). The primers were ordered from TAG Copenhagen AS (Copenhagen, Denmark). qPCR reactions were performed in duplicate or triplicate for each rat for each gene and the total reaction volume was 10 μl. Results were normalised to *Gapdh* (Glyceraldehyde-3-phosphate dehydrogenase) using the comparative C_T_ (2^−ΔΔcT^) method (Schmittgen and Livak, 2008).

**TABLE 1:** qPCR primer sequences for the gene expression assay.

Target genes	Primer sequence	Accession number
*Dnmt1*	Forward: AAC​GGA​ACA​CTC​TCT​CTC​ACT​CA	NM_053354
	Reverse: TCA​CTG​TCC​GAC​TTG​CTC​CTC	
*Dnmt3a*	Forward: CAG​CGT​CAC​ACA​GAA​GCA​TAT​CC	NM_001003958
	Reverse: GGT​CCT​CAC​TTT​GCT​GAA​CTT​GG	
*Dnmt3b*	Forward: GAA​TTT​GAG​CAG​CCC​AGG​TTG	NM_001003959
	Reverse: TGA​AGA​AGA​GCC​TTC​CTG​TGC​C	
*Tet1*	Forward: TGT​CAC​CTG​TTG​CAT​GGA​TT	NM_001107643
	Reverse: TTG​GAT​CTT​GGC​TTT​CAT​CC	
*Tet2*	Forward: GAG​GAG​CAG​AAG​GAA​GCA​AG	XM_227694.9
	Reverse: CAC​CGT​AGC​AGA​ACA​GGA​AC	
*Tet3*	Forward: CAG​GGA​CCA​AGC​AAC​AGA​AC	XM_032906253.1
	Reverse: AGG​GTG​TGA​GAG​GAA​AGA​GG	
*Gapdh*	Forward: TGC​ACC​ACC​AAC​TGC​TTA​GC	NM_017008
	Reverse: GGC​ATG​GAC​TGT​GGT​CAT​GAG	
*Kor*	Forward: AGC​TCT​TGG​TTC​CCC​AAC​TG	NM_017167
	Reverse: CAC​CAC​AGA​GTA​GAC​AGC​GG	
*Pdyn*	Forward: CCT​GTC​CTT​GTG​TTC​CCT​GT	NM_019374
	Reverse: AGA​GGC​AGT​CAG​GGT​GAG​AA	

### DNMT and TET Activity Measurements

Nuclear proteins were extracted from NAc tissue according to the manufacturer’s protocol (Nuclear Extraction kit; Abcam, Cambridge, United Kingdom), and 5 μg of nuclear protein extracts were used for assays. DNMT activity was determined using an EpiQuik DNMT Activity/Inhibition Assay Ultra Kit and TET activity was measured using Epigenase 5mC-Hydroxylase TET Activity/Inhibition assay kit (Epigentek Group, Brooklyn, NY, United States) according to manufacturer’s instructions. DNMT and TET activity levels (OD/h/mg) were calculated according to the manufacturer’s protocol.

### Quantification of Global DNA Methylation and Hydroxymethylation Levels

Genomic DNA was extracted from NAc tissue using DNeasy Blood & Tissue Kit (Qiagen, Hilden, Germany) according to the manufacturer’s protocol and 100 ng of DNA were used for further assays. Global DNA methylation and hydroxymethylation analysis were performed using a Global DNA Methylation Assay Kit (Abcam, Cambridge, United Kingdom) and a Global DNA Hydroxymethylation Assay Kit (Abcam, Cambridge, United Kingdom) according to the manufacturer’s instructions. The percentage of methylated DNA (5mC%) and hydroxymethylated DNA (5hmC%) in total DNA was calculated according to the manufacturer’s protocol and formula.

### DNA Isolation and 5hmC and 5mC Analyses at the Specific CCGG Site

Genomic DNA from new NAc tissue were extracted as above. The 5hmC and 5mC levels at CCGG sites in *Dnmt3a, Kor, Pdyn* promoter regions were detected according to the manufacturer’s instructions using the EpiMark 5hmC and 5mC Analysis Kit (New England Biolabs, MA, United States). Briefly, 5 μg of NAc genomic DNA were treated with T4 Phage b-glucosyltransferase and incubated at 37°C for 16 h. Glucosylated genomic DNA was digested either with 100 U of MspI or 50 U of HpaII or no enzyme (negative control) at 37°C for 5 h. Digestion was terminated using Proteinase K at 40°C for 30 min and inactivated at 95°C for 10 min. Real-time PCR was then performed on 1 μl of the glycosylated/digested DNA using site-specific primers listed in Table 2. The percentages of the 5hmC, 5mC, and cytosine of the inner C at the site-specific CCGG were calculated using the EpiMark comparative C_t_ method.

**TABLE 2:** qPCR primer sequences for the gene promoter region assay.

Target genes	Primer sequence	Accession number
*Dnmt3a*	Forward: GAC​ACG​CTA​CAC​CTT​CGA​GT	NM_001003958
	Reverse: CCC​AGT​CTC​ACC​AAC​ACC​TC	
*Kor*	Forward: CTC​TCC​TGT​GGA​CAT​GGT​GT	NM_017167
	Reverse: ACA​GCC​TAA​ATG​TCC​TTG​TCA​G	
*Pdyn*	Forward: GGA​AGA​CAG​CCT​GAG​ACA​CA	NM_019374
	Reverse: GCG​GCT​TTG​CGT​GAT​TAA​AC	

### Statistical Analysis

All data are expressed as the mean ± standard error of the mean (SEM). The significance level was established at 0.05 and a *p*-value of less than 0.05 was considered statistically significant. One-way ANOVA with repeated measures followed by Tukey’s multiple comparisons test were used for comparisons using GraphPad Prism 9 software (GraphPad, San Diego, CA, United States).

## Results

### The Effect of Intermittent Alcohol Exposure on *Dnmt* and *Tet* Family Gene Expression in the NAc

**FIGURE 1:**
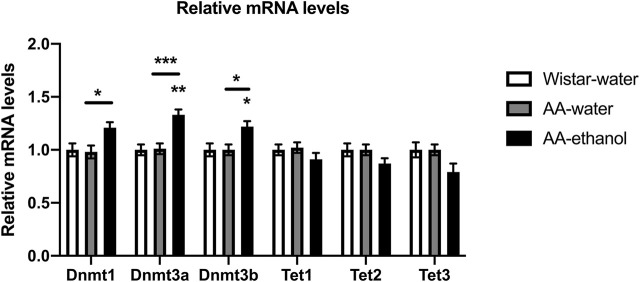
Impact of intermittent alcohol exposure on *Dnmt1, Dnmt3a, Dnmt3b, Tet1, Tet2, and Tet3* expression levels in the NAc. One-way ANOVA followed by Tukey’s multiple comparisons test; ***p < 0.001, **p < 0.01, *p < 0.05 compared with the Wistar-water group; *n* = 5–7. Error bars indicate SEM.

First, we used qPCR to evaluate the effect of intermittent alcohol exposure on expression levels of *Dnmt* and *Tet* genes in NAc. Our data showed (Figure 1), that ethanol treatment increased significantly the mRNA levels of *Dnmts* in the NAc after alcohol exposure (one-way ANOVA main effect of the group: *Dnmt1*, F_(2,16)_ = 5.324, *p* = 0.0169; *Dnmt3a*, F_(2,16)_ = 14.30, *p* = 0.0003; *Dnmt3b*, F_(2,16)_ = 6.070, *p* = 0.0109; followed by Tukey’s post-hoc test; *Dnmt1*, *p* < 0.05 AA-water vs. AA-ethanol; mean ± SEM values: Wistar-water 1.00 ± 0.06; AA-water 0.98 ± 0.06; AA-ethanol 1.21 ± 0.05; *Dnmt3a*, *p* < 0.01, *p* < 0.001 AA-water vs. AA-ethanol; mean ± SEM values: Wistar-water 1.00 ± 0.05; AA-water 1.01 ± 0.05; AA-ethanol 1.33 ± 0.05; *Dnmt3b*, *p* < 0.05, *p* < 0.05 AA-water vs. AA-ethanol; mean ± SEM values: Wistar-water 1.00 ± 0.06; AA-water 1.00 ± 0.05; AA-ethanol 1.22 ± 0.05, *n* = 5–7). A trend towards a reduction in mRNA levels of *Tet* genes in the NAc after ethanol treatment was observed, but these changes did not reach statistical significance (*Tet1*, mean ± SEM values: Wistar-water 1.00 ± 0.05; AA-water 1.02 ± 0.05; AA-ethanol 0.91 ± 0.06; *Tet2*, mean ± SEM values: Wistar-water 1.00 ± 0.06; AA-water 1.00 ± 0.05; AA-ethanol 0.87 ± 0.05; *Tet3*, mean ± SEM values: Wistar-water 1.00 ± 0.07; AA-water 1.00 ± 0.05; AA-ethanol 0.79 ± 0.08, *n* = 5–7).

### Changes in DNMT and TET Enzyme Activity in Response to Ethanol Exposure

**FIGURE 2:**
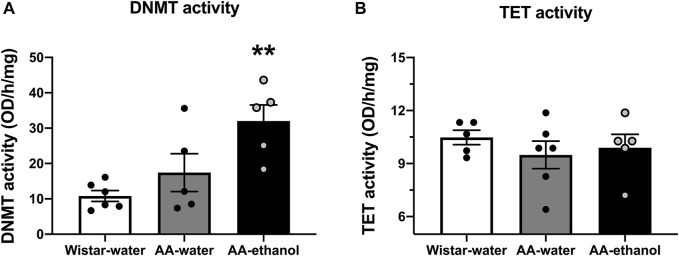
Impact of intermittent alcohol exposure on DNMT **(A)** and TET **(B)** enzyme activity levels in the NAc. One-way ANOVA followed by Tukey’s multiple comparisons test; **p < 0.01, compared with the Wistar-water group; *n* = 5–6. Error bars indicate SEM.

As the transcription levels of *Dnmt1, Dnmt3a,* and *Dnmt3b* were altered following ethanol exposure, we measured the activity of DNMT and TET enzymes in the NAc. As illustrated in Figure 2A, ethanol intake increased DNMT activity (F_(2, 13)_ = 7.757, *p* = 0.0061; mean ± SEM values: Wistar-water 10.82 ± 1.53; AA-water 17.42 ± 5.37; AA-ethanol 32.04 ± 4.53, *n* = 5–6) in AA-ethanol compared with Wistar-water group (*p* < 0.01). No significant changes were observed in TET activity (Figure 2B, F_(2,13)_ = 0.5209, *p* = 0.6059; mean ± SEM values: Wistar-water 10.48 ± 0.41; AA-water 9.49 ± 0.78; AA-ethanol 9.89 ± 0.76, *n* = 5–6) in response to ethanol intake. These results are similar to our qPCR observations where ethanol altered *Dnmt* genes and did not affect *Tet* genes.

### Changes in Global 5mC and 5hmC Levels in Response to Ethanol Treatment

**FIGURE 3:**
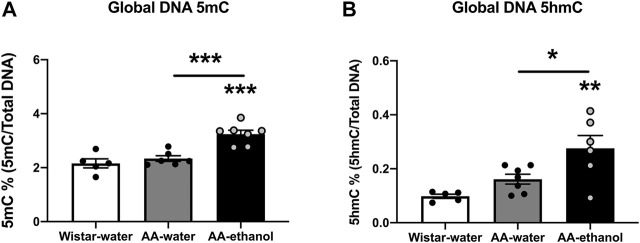
Global DNA methylation (5mC%) **(A)** and hydroxymethylation (5hmC %) **(B)** levels in the NAc. One-way ANOVA followed by Tukey’s multiple comparisons test; ***p < 0.001, **p < 0.01, *p < 0.05, compared with the Wistar-water group; *n* = 5–7. Error bars indicate SEM.

Next, we analysed global DNA 5mC and 5hmC levels in the NAc in response to ethanol treatment. We observed a significant increase in the relative percentage of 5mC (Figure 3A) in the AA-ethanol group compared with the AA-water and Wistar-water groups (one-way ANOVA main effect of the group: F_(2,15)_ = 17.98, *p* = 0.0001; followed by Tukey’s post-hoc test; *p* < 0.001 compared with Wistar-water group, *p* < 0.001 AA-water vs. AA-ethanol; mean ± SEM values: Wistar-water 2.16 ± 0.17; AA-water 2.34 ± 0.11; AA-ethanol 3.24 ± 0.14, *n* = 5–7). Interestingly, the global level of 5hmC was also increased in the AA-ethanol group (Figure 3B, one-way ANOVA main effect of the group: F_(2,15)_ = 8.372, *p* = 0.0036; followed by Tukey’s post-hoc test; *p* < 0.01compared with Wistar-water group, *p* < 0.05 AA-water vs. AA-ethanol; mean ± SEM values: Wistar-water 0.10 ± 0.01; AA-water 0.16 ± 0.02; AA-ethanol 0.28 ± 0.05, *n* = 5–7). These data demonstrate that ethanol treatment increases both DNA methylation and hydroxymethylation in the NAc.

### The Effect of Intermittent Alcohol Exposure on *Pdyn* and *Kor* Family Gene Expression in the NAc

**FIGURE 4:**
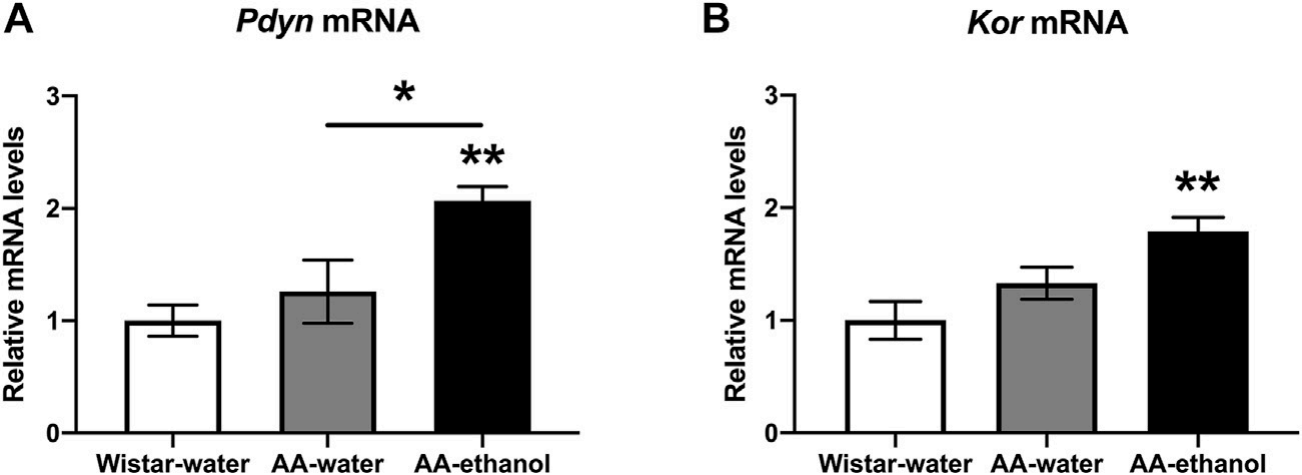
Impact of intermittent alcohol exposure on *Pdyn* **(A)** *Kor* **(B)** expression levels in the NAc. One-way ANOVA followed by Tukey’s multiple comparisons test; **p < 0.01, *p < 0.05 compared with the Wistar-water group; *n* = 5–7. Error bars indicate SEM.

Next, we studied the effect of ethanol treatment on mRNA levels of selected candidate genes related to ethanol addiction in NAc. We selected prodynorphin (*Pdyn*) and kappa opioid receptor (*Kor*) because these candidate genes have been demonstrated to be affected by ethanol (Uhari-Väänänen et al., 2018, 2019)**.** The gene expression study revealed (Figure 4A) a significant increase in the levels of *Pdyn* in the AA-ethanol group (one-way ANOVA main effect of the group: F_(2,14)_ = 7.609, *p* = 0.0058; followed by Tukey’s post-hoc test; *p* < 0.01, *p* < 0.05 AA-water vs. AA-ethanol; mean ± SEM values: Wistar-water 1.00 ± 0.10; AA-water 1.26 ± 0.28; AA-ethanol 2.07 ± 0.13, *n* = 5–7). Kor mRNA (Figure 4B) levels were similarly increased in the AA-ethanol group (*p* < 0.01) after alcohol exposure (one-way ANOVA main effect of the group: F_(2,15)_ = 6.884, *p* = 0.0076; mean ± SEM values: Wistar-water 1.00 ± 0.10; AA-water 1.33 ± 0.14; AA-ethanol 1.79 ± 0.13, *n* = 5–7).

### Changes in the Promoter Region Methylation and Levels of Selected Candidate Genes After Ethanol Treatment

**FIGURE 5:**
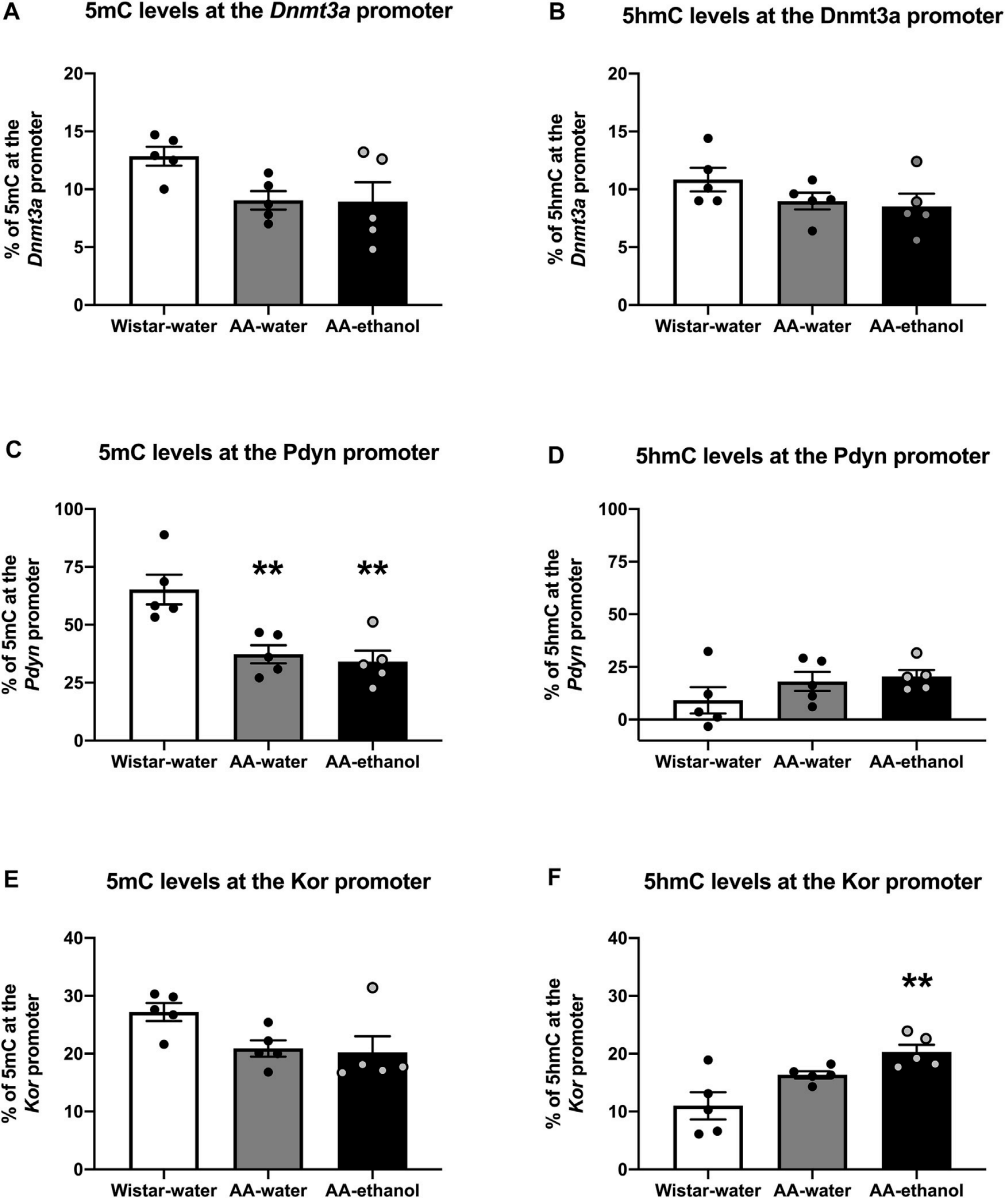
Promoter region methylation (5mC%) **(A,C,E)** and hydroxymethylation (5hmC %) **(B,D,F)** levels in the NAc. One-way ANOVA followed by Tukey’s multiple comparisons test; **p < 0.01, compared with the Wistar-water group; *n* = 5–7. Error bars indicate SEM.

Altered global changes in DNA methylation and hydroxymethylation could cause changes at certain gene promoters enriched with CpG islands and consequently influence the expression of these genes. In this work, we evaluated the transcription of several genes involved in opioid- and dopaminergic systems and related to drug addiction (Supplementary Figures S1, S2). We chose to investigate promoter methylation status of the *Dnmt3a*, *Pdyn,* and *Kor* genes (Figure 5) following ethanol treatment as we observed a change in the mRNA levels of these genes. Our data showed that in the *Dnmt3a* promoter, 5mC% (Figure 5A and Supplementary Table S2) and 5hmC% (Figure 5B and Supplementary Table S2), were slightly reduced in AA-water and AA-ethanol groups (one-way ANOVA main effect of the group: 5mC%, F_(2, 12)_ = 3.623, *p* = 0.0588; mean ± SEM values: Wistar-water 12.87 ± 0.82; AA-water 9.04 ± 0.81; AA-ethanol 8.93 ± 1.67, *n* = 5–7; 5hmC%, F_(2,12)_ = 1.625, *p* = 0.2375; mean ± SEM values: Wistar-water 10.84 ± 1.03; AA-water 8.96 ± 0.72; AA-ethanol 8.51 ± 1.11, *n* = 5–7), but these changes did not reach statistical significance.

Regarding the *Pdyn* promoter methylation status we observed a significant reduction in 5mC% (Figure 5C and Supplementary Table S3) in AA-water and AA-ethanol groups (one-way ANOVA main effect of the group: F_(2, 12)_ = 11.06, *p* = 0.0019; followed by Tukey’s post-hoc test *p* < 0.01 Wistar-water vs. AA-water, *p* < 0.01 Wistar-water vs. AA-ethanol; mean ± SEM values: Wistar-water 65.23 ± 6.43; AA-water 37.26 ± 3.92; AA-ethanol 34.11 ± 4.77, *n* = 5–7). We also observed a slight increase in promoter 5hmC% (Figure 5D and Supplementary Table S3) in AA-water and AA-ethanol groups (one-way ANOVA main effect of the group: F_(2,12)_ = 1.526, *p* = 0.2568; mean ± SEM values: Wistar-water 9.19 ± 6.33; AA-water 18.11 ± 4.53; AA-ethanol 20.48 ± 3.09, *n* = 5–7), but due to variability between animals, these changes did not reach statistical significance.

Finally, we analysed *Kor* promoter methylation and observed that 5mC% was reduced slightly (Figure 5E and Supplementary Table S4) in AA-water and AA-ethanol groups (one-way ANOVA main effect of the group: F_(2, 12)_ = 3.621, *p* = 0.0581; mean ± SEM values: Wistar-water 27.21 ± 1.55; AA-water 20.91 ± 1.42; AA-ethanol 20.18 ± 2.81, *n* = 5–7) whereas 5hmC% was increased (Figure 5F and Supplementary Table S4) in the AA-ethanol group (one-way ANOVA main effect of the group: F_(2,12)_ = 8.793, *p* = 0.0045; followed by Tukey’s post-hoc test *p* < 0.01 Wistar-water vs. AA-ethanol, mean ± SEM values: Wistar-water 11.01 ± 2.35; AA-water 16.35 ± 0.62; AA-ethanol 20.30 ± 1.24, *n* = 5–7).

## Discussion

In this work, we investigated whether intermittent ethanol exposure affects the expression and enzymatic activity of the epigenetic DNA modifiers DNMT and TET using the alcohol-preferring rat model. Previously, we have shown that addictive substances and environmental factors, may affect the transcription of epigenetic DNA modifiers in different brain regions (Anier et al., 2018; Urb et al., 2020). Thus, it can be assumed that ethanol causes similar epigenetic changes in different regions of the brain. As the NAc plays a central role in the mechanisms of drug addiction, we focused our study on this area.

We report three key findings. First, we show that intermittent ethanol exposure increased significantly the mRNA levels of DNMTs and these changes were associated with significant increases in enzymatic activity in AA-ethanol rats compared with control rats in the NAc. No significant changes were observed in TET mRNA levels or enzymatic activity. Second, we demonstrate a significant increase in the relative percentage of global methylation and hydroxymethylation in the AA-ethanol group compared with the control groups. Finally, our selected gene expression and promoter methylation status analysis revealed a significant increase in the mRNA levels of *Pdyn*, *Kor,* and *Dnmt3a*; however, we failed to convincingly demonstrate that the aberrant DNA methylation in promoter areas are responsible for the changed gene expression of these genes.

Despite a decade of research, there is no consensus on alcohol-induced brain DNA methylation in experimental animal models. Previous studies in rodents have been suggested that chronic ethanol consumption induces DNA hypomethylation with histone acetylation (Mahadev and Vemuri, 1998; Berkel and Pandey, 2017). Subsequent studies in experimental animal models suggests that chronic ethanol administration tends to increase DNA methylation in various parts of the brain (Warnault et al., 2013; Barbier et al., 2015). In this study, we found that intermittent alcohol drinking in AA-rats increased significantly *Dnmt3a* and to a lesser extent both *Dnmt1* and *Dnmt3b* mRNA levels, and the increase in expression of these genes was associated with increased DNMT enzymatic activity in NAc. Our data are consistent with previously published results, for instance, excessive alcohol drinking increased *Dnmt1* expression and reduced histone H4 acetylation in NAc of rodents and reducing DNA methylation by inhibiting the activity of DNMT with systemic administration of 5-azacitidine prevented excessive alcohol use in mice (Warnault et al., 2013). Similarly, another study of NAc and medial prefrontal cortex (mPFC) from rats following weeks of abstinence from ethanol showed a significant increase in *Dnmt1* expression and in global DNA methylation that was associated with a downregulation of a cluster of synaptic genes specifically within mPFC neurons (Barbier et al., 2015).

Only a few reports have been described the aberrant DNA methylation in the alcohol-preferred rat model. Qiao et al. (2017) demonstrated that, both of the mRNA and protein levels of DNMT3A and -3B in mPFC were upregulated after 35 days of alcohol exposure and this upregulation could be reversed by 5-Aza-2′-deoxycytidine treatment. Recently, Cui et al. (2020) showed, using the reduced representative bisulfite sequencing technology, that the methylation level of promoter region in the mPFC of rats exposed to chronic alcohol was significantly increased. In addition, chronic alcohol exposure increased the mRNA and protein levels of DNMT3B and methyl CpG binding protein 2. However, short term alcohol exposure did not affect their expression. Therefore, based on previous reports and our results, accumulating data indicate that repeated ethanol exposure increases DNMTs expression in various brain regions in different animal models of AUD.

There are fewer data on alcohol-induced DNA demethylation in brain and other tissues. Finegersh et al. (2015) demonstrated that repeated intermittent alcohol exposure increased *Tet1* mRNA expression in the NAc but not in cortex in a mouse AUD model. However, in a recently published study, Ji et al. (2019) found that ethanol exposure to liver cells reduces *Tet1* and 5hmC formation is involved in hepatocyte apoptosis in alcoholic liver disease progression. We found no changes in *Tet1-3* mRNA levels or DNMT activity in NAc in the rat AA model. The effect of repeated ethanol administration on TET gene expression and enzymatic activity in different brain regions has not been previously studied in a rat model. Thus, results suggest that the effect of repeated ethanol on TET gene expression and enzymatic activity is more variable than DNMTs expression and may depend on the species of animal and the tissue under examination.

The data collected suggest that both DNMTs and TETs may function as epigenetic editors in the mechanisms of drug addiction induced by substance of abuse (Saha et al., 2021). The exact mechanism underlying ethanol-mediated changes in DNMT and TET gene expressions is poorly understood. It has been suggested that ethanol alters receptors (glutamate, dopamine, GABA) and ion channels which subsequently alter intracellular signalling cascades leading to the activation or inhibition of transcription factors and other nuclear proteins, eventually changing the expression of DNA modifying enzymes (Nestler, 2014). Taken together, our results from AA-ethanol rats suggest that repeated intermittent exposure to ethanol increases DNMT activity and it may be one of the molecular mechanisms for aberrant DNA methylation.

Changes in DNMT enzymatic activity implies possible downstream changes in percent global 5mC. To gain additional information on aberrant DNA methylation induced by repeated alcohol drinking, the global levels of 5mC and 5hmC in the NAc were measured. Our results demonstrated an increase in percent 5mC in AA-ethanol rats compared with control groups. These changes concur with our data showing increased DNMT enzymatic activity in NAc in AA-ethanol rats.

Previous studies in rodent models of AUD have not studied the global level of 5mC and 5hmC in the NAc after intermittent administration of ethanol. Surprisingly, we also found a statistically significant increase in global 5hmC levels. We speculate that an increase in 5mC (a precursor of 5hmC) leads to increases in 5hmC even if TET enzymatic activity is not changed. Thus, an increase in 5hmC levels may be a compensatory process to ensure optimal DNA demethylation in the cell. Changes in the global levels of 5mC and 5hmC also suggest that a new DNA methylation and demethylation balance develops following repeated ethanol exposure in the NAc. We have found similar phenomena also after repeated psychostimulant exposure (Anier et al., 2018). Therefore, our results support the findings that prolonged ethanol drinking increases the extent of DNA methylation at the whole-genome level and that these changes may underlie altered gene expression changes in the NAc.

In addition to the changes in ethanol-induced global methylation, we were interested in whether repeated ethanol drinking also affects the methylation state of promoters of selected genes in the AA rat model. There is currently no consensus on genes that could serve as marker genes in AUD models. Therefore, we selected candidate genes that have been studied in various alcohol models.

We examined the transcription of several genes from dopaminergic and opioidergic systems (Figure 4, Supplementary Figures S1, S2) previously associated with the AUD. In addition, we also analyzed DNA methyltransferases. There were no changes in *Dat* gene, but we found significant increases in *Drd1*, *Drd2*, *Kor*, *Pdyn,* and *DNMT3a* mRNA levels in AA-ethanol rats. The results of the methylation status in the promoter areas of selected genes were more complex. The *Pdyn* promoter region under study was significantly hypomethylated, while in the *Kor* and *Dnmt3a* promoter regions we saw a trend towards hypomethylation, but it did not reach statistical significance. Previous studies of promoter methylation status had revealed several genes that are affected by the interplay between alcohol and epigenetic regulation and that may play a role in alcohol addiction (Basavarajappa and Subbanna, 2016). Some of these genes exhibited hypermethylated promoters with others showing an opposite trend, suggesting that alcohol’s effects on DNA methylation are diverse and may be affected by numerous factors, including developmental stage, functional state of the cell, and specific gene targets in specific cell types. Using prenatal ethanol exposure model, Wille-Bille and colleagues demonstrated (Wille-Bille et al., 2018) upregulation of *Pdyn* and *Kor* mRNA levels in the ventral tegmental area (VTA) in infant and adolescent rats and *Kor* mRNA levels in the prefrontal cortex in infant rats. The changes in gene expression in the VTA were accompanied by a reduction of DNA methylation at the *Pdyn* gene promoter, and by a reduction of DNA methylation at the *Kor* gene promoter. These results suggest that different patterns of alcohol consumption in experimental animals may similarly affect the methylation and transcription of *Pdyn* and *Kor* gene promoters.

However, our results demonstrate a discrepancy between increased DNMT expression, increased global methylation and increased transcription of the selected genes in the NAc. The results of our selected genes (*Kor*, *Dnmt3a*) showed that the increase in transcription after repeated alcohol consumption in AA rats was only partially explained by a 5mC decrease in promoter, suggesting that other regulatory mechanisms may be involved. Accumulating data suggest that the DNA methylation and histone modification interact and influence each other and together may fine-tune gene expression (Cedar and Bergman, 2009; Krishnan et al., 2014). Alternatively, promoter regions selected for methylation status analysis were not representative. The possible weakness of 5hmC and 5mC detection methods at the specific CCGG is that it evaluates the change in methylation patterns in a very short region of the promoter, which makes it not necessarily representative.

Interestingly, the changes in the 5hmC level of the selected gene promoters were variable. This may indicate that after repeated alcohol administration a new balance between 5mC and 5hmC levels may be formed in the promoter region of some genes (e.g., *Kor*, *Pdyn*). However, no definitive conclusions about 5hmC changes can be drawn from the analysis of the three genes.

Finally, the results of our study indicate that the epigenetic changes also contribute to the AA-rat model susceptibility to ethanol drinking rather than being a pure response to alcohol exposure. This speculation is supported by the differences in DNMT enzymatic activity and the marker genes promoter methylation in AA-water rats compared to Wistar-water rats. The underlying mechanism for these epigenetic changes is unclear, but we hypotheses that the stress level of an individual rat in the early stages of development may affect epigenetic mechanisms of basal level and some of these epigenetic changes may persist into adulthood (Anier et al., 2014) and cause the higher-level ethanol consumption in adulthood. Further studies should elucidate possible mechanisms of ethanol sensitivity in AA-rats.

## Conclusion

Our results, using alcohol preferring AA-rat model, support previous findings that repeated alcohol exposure tends to increase DNMT activity and may induce the aberrant DNA methylation in the NAc. The aberrant DNA methylation together with other epigenetic mechanism, such as histone modification can be the specific molecular mechanisms mediating dependence-induced neuroadaptations induced by alcohol consumption.

## Data Availability

The original contributions presented in the study are included in the article/Supplementary Material, further inquiries can be directed to the corresponding author.
