# Supplementary material for: Repeated Ethanol Exposure Alters DNA Methylation Status and Dynorphin/Kappa-Opioid Receptor Expression in Nucleus Accumbens of Alcohol-Preferring AA Rats
Source: Front Genet. 2021 Nov 24;12:750142. doi: 10.3389/fgene.2021.750142 (PMC8652212; doi:10.3389/fgene.2021.750142)
Supplement: Supplementary file 1 [file Table1.docx]

# SUPPLEMENTARY MATERIALS

**Supplementary Table 1.** **Additional qPCR primer sequences for gene expression assay**

| **Target genes** | **Primer sequence** |
| --- | --- |
| ***Npy*** | Forward: ATGCTAGGTAACAAACG  Reverse: ATGTAGTGTCGCAGAG |
| ***Penk*** | Forward: CCTCCGACCTGCTGAAAGAG  Reverse: CCCCATACCTCTTGCTCGTG |
| ***Oprl1*** | Forward: TGGGGAACTGCCTCGTCATGT  Reverse: TTCCCAAATGGCCAGAAGCCCA |
| ***Oprd1*** | Forward: TCGTCCGGTACACTAAGCTG  Reverse: GGCCACGTTTCCATCAGGTA |
| ***Oprm1*** | Forward: AGTTCTGCATCCCAACCTCG  Reverse: GCCTCCAGATTTTCTAGCTGGT |
| ***Pnoc*** | Forward: TGCAGCACCTGAAGAGAATG  Reverse: CAACTTCCGGGCTGACTTC |
| ***Pomc*** | Forward: AGGTTAAGGAGCAGTGACTAAG  Reverse: CGTCTATGGAGGTCTGAAGC |
| ***Drd1*** | Forward: CCTTCGATGTGTTTGTGTGG  Reverse: GGGCAGAGTCTGTAGCATCC |
| ***Drd2*** | Forward: TCCTGTCCTTCACCATCTCC  Reverse: GACCAGCAGAGTGACGATGA |
| ***Dat*** | Forward: CCCGGCTAAAGAGTCCAATGCT  Reverse: GGTGTCTGTGGCGGGTTGAT |
| ***Ppp1cc*** | Forward: ACACTGCCTAGTGGGACTTG  Reverse: AATCAAAATGGAGAAGGGAGGGC |
| ***A2ar*** | Forward: AGTCAGAAAGACGGGAAC  Reverse: CAGTAACACGAACGCAA |
