# Supplementary material for: Repeated Ethanol Exposure Alters DNA Methylation Status and Dynorphin/Kappa-Opioid Receptor Expression in Nucleus Accumbens of Alcohol-Preferring AA Rats
Source: Front Genet. 2021 Nov 24;12:750142. doi: 10.3389/fgene.2021.750142 (PMC8652212; doi:10.3389/fgene.2021.750142)
Supplement: Supplementary file 2 [file Table2.docx]

**Supplementary Table 2**

***Dnmt3a* promoter region methylation (5-mC%) and hydroxymethylation (5-hmC %)**

| **Wistar-Water** |  |  | **%** |  |  | **Group total** | **%** | stdev | SEM |
| --- | --- | --- | --- | --- | --- | --- | --- | --- | --- |
| **W-W36** | ChmCGG% | 0,144 | **14,4** | 5-hmC |  | **W-W group** |  |  |  |
|  | CmCGG% | 0,129 | **12,9** | 5-mC |  | 5-hmC | **10,84** | 2,29 | 1,03 |
| **W-W37** | ChmCGG% | 0,117 | **11,7** | 5-hmC |  | 5-mC | **12,87** | 1,8 | 0,82 |
|  | CmCGG% | 0,125 | **12,5** | 5-mC |  |  |  |  |  |
| **W-W38** | ChmCGG% | 0,090 | **9,0** | 5-hmC |  |  |  |  |  |
|  | CmCGG% | 0,142 | **14,2** | 5-mC |  |  |  |  |  |
| **W-W39** | ChmCGG% | 0,101 | **10,1** | 5-hmC |  |  |  |  |  |
|  | CmCGG% | 0,147 | **14,7** | 5-mC |  |  |  |  |  |
| **W-W40** | ChmCGG% | 0,090 | **9,0** | 5-hmC |  |  |  |  |  |
|  | CmCGG% | 0,100 | **10,0** | 5-mC |  |  |  |  |  |
|  |  |  |  |  |  |  |  |  |  |
| **AA-Water** |  |  | **%** |  |  | **Group total** | **%** | stdev | SEM |
| **AA-W24** | ChmCGG% | 0,108 | **10,8** | 5-hmC |  | **AA-W** |  |  |  |
|  | CmCGG% | 0,114 | **11,4** | 5-mC |  | 5-hmC | **8,96** | 1,61 | 0,72 |
| **AA-W25** | ChmCGG% | 0,064 | **6,4** | 5-hmC |  | 5-mC | **9,04** | 1,8 | 0,81 |
|  | CmCGG% | 0,087 | **8,7** | 5-mC |  |  |  |  |  |
| **AA-W26** | ChmCGG% | 0,091 | **9,1** | 5-hmC |  |  |  |  |  |
|  | CmCGG% | 0,078 | **7,8** | 5-mC |  |  |  |  |  |
| **AA-W27** | ChmCGG% | 0,096 | **9,6** | 5-hmC |  |  |  |  |  |
|  | CmCGG% | 0,070 | **7,0** | 5-mC |  |  |  |  |  |
| **AA-W28** | ChmCGG% | 0,090 | **9,0** | 5-hmC |  |  |  |  |  |
|  | CmCGG% | 0,103 | **10,3** | 5-mC |  |  |  |  |  |
|  |  |  |  |  |  |  |  |  |  |
| **AA-Ethanol** |  |  | **%** |  |  | **Group total** | **%** | stdev | SEM |
| **AA-E8** | ChmCGG% | 0,056 | **5,6** | 5-hmC |  | **AA-E** |  |  |  |
|  | CmCGG% | 0,126 | **12,6** | 5-mC |  | 5-hmC | **8,51** | 2,47 | 1,11 |
| **AA-E9** | ChmCGG% | 0,089 | **8,9** | 5-hmC |  | 5-mC | **8,93** | 3,7 | 1,67 |
|  | CmCGG% | 0,048 | **4,8** | 5-mC |  |  |  |  |  |
| **AA-E10** | ChmCGG% | 0,078 | **7,8** | 5-hmC |  |  |  |  |  |
|  | CmCGG% | 0,075 | **7,5** | 5-mC |  |  |  |  |  |
| **AA-E11** | ChmCGG% | 0,124 | **12,4** | 5-hmC |  |  |  |  |  |
|  | CmCGG% | 0,132 | **13,2** | 5-mC |  |  |  |  |  |
| **AA-12** | ChmCGG% | 0,079 | **7,9** | 5-hmC |  |  |  |  |  |
|  | CmCGG% | 0,065 | **6,5** | 5-mC |  |  |  |  |  |
