# Supplementary material for: Repeated Ethanol Exposure Alters DNA Methylation Status and Dynorphin/Kappa-Opioid Receptor Expression in Nucleus Accumbens of Alcohol-Preferring AA Rats
Source: Front Genet. 2021 Nov 24;12:750142. doi: 10.3389/fgene.2021.750142 (PMC8652212; doi:10.3389/fgene.2021.750142)
Supplement: Supplementary file 3 [file Table3.docx]

| **Wistar-Water** | |  | | |  | | | | **%** | | |  | | **Group total** | | | **%** | | | stdev | | | | SEM | |
| --- | --- | --- | --- | --- | --- | --- | --- | --- | --- | --- | --- | --- | --- | --- | --- | --- | --- | --- | --- | --- | --- | --- | --- | --- | --- |
| **W-W36** | | ChmCGG% | | | 0,324 | | | | **32,4** | | | 5-hmC | | **W-W group** | | |  | | |  | | | |  | |
|  | | CmCGG% | | | 0,533 | | | | **53,3** | | | 5-mC | | 5-hmC | | | **9,19** | | | 14,16 | | | | 6,33 | |
| **W-W37** | | ChmCGG% | | | 0,036 | | | | **3,6** | | | 5-hmC | | 5-mC | | | **65,23** | | | 14,4 | | | | 6,43 | |
|  | | CmCGG% | | | 0,889 | | | | **88,9** | | | 5-mC | |  |  |  |  |  |  |  |  |  |  |  |  |
| **W-W38** | | ChmCGG% | | | 0,011 | | | | **1,1** | | | 5-hmC | |  | | |  | | |  | | | |  | |
|  | | CmCGG% | | | 0,582 | | | | **58,2** | | | 5-mC | |  | | |  | | |  | | | |  | |
| **W-W39** | | ChmCGG% | | | -0,033 | | | | **-3,3** | | | 5-hmC | |  | | |  | | |  | | | |  | |
|  | | CmCGG% | | | 0,686 | | | | **68,6** | | | 5-mC | |  | | |  | | |  | | | |  | |
| **W-W40** | | ChmCGG% | | | 0,120 | | | | **12,0** | | | 5-hmC | |  | | |  | | |  | | | |  | |
|  | | CmCGG% | | | 0,571 | | | | **57,1** | | | 5-mC | |  | | |  | | |  | | | |  | |
|  | |  | | |  | | | |  | | |  | |  | | |  | | |  | | | |  | |
| **AA-Water** | |  | | |  | | | | **%** | | |  | | **Group total** | | | **%** | | | stdev | | | | SEM | |
| **AA-W24** | | ChmCGG% | | | 0,112 | | | | **11,2** | | | 5-hmC | | **AA-W group** | | |  | | |  | | | |  | |
|  | | CmCGG% | | | 0,457 | | | | **45,7** | | | 5-mC | | 5-hmC | | | **18,11** | | | 10,13 | | | | 4,53 | |
| **AA-W25** | | ChmCGG% | | | 0,163 | | | | **16,3** | | | 5-hmC | | 5-mC | | | **37,26** | | | 8,8 | | | | 3,92 | |
|  | | CmCGG% | | | 0,467 | | | | **46,7** | | | 5-mC | |  | | |  | | |  | | | |  | |
| **AA-W26** | | ChmCGG% | | | 0,061 | | | | **6,1** | | | 5-hmC | |  | | |  | | |  | | | |  |  |
|  | | CmCGG% | | | 0,360 | | | | **36,0** | | | 5-mC | |  | | |  | | |  | | | |  |  |
| **AA-W27** | | ChmCGG% | | | 0,292 | | | | **29,2** | | | 5-hmC | |  | | |  | | |  | | | |  | |
|  | | CmCGG% | | | 0,271 | | | | **27,1** | | | 5-mC | |  | | |  | | |  | | | |  | |
| **AA-W28** | | ChmCGG% | | | 0,278 | | | | **27,8** | | | 5-hmC | |  | | |  | | |  | | | |  | |
|  | | CmCGG% | | | 0,309 | | | | **30,9** | | | 5-mC | |  | | |  | | |  | | | |  | |
|  | |  |  | | |  | |  | |  | | | |  |  | | | | | |  | |  |  |  |
| **AA-Ethanol** | |  | | |  | | | | **%** | | |  | | **Group total** | | | **%** | | | stdev | | | | SEM | |
| **AA-E8** | | ChmCGG% | | | 0,317 | | | | **31,7** | | | 5-hmC | | **AA-E group** | | |  | | |  | | | |  | |
|  | | CmCGG% | | | 0,226 | | | | **22,6** | | | 5-mC | | 5-hmC | | | **20,48** | | | 6,90 | | | | 3,09 | |
| **AA-E9** | | ChmCGG% | | | 0,201 | | | | **20,1** | | | 5-hmC | | 5-mC | | | **34,11** | | | 10,7 | | | | 4,77 | |
|  | | CmCGG% | | | 0,513 | | | | **51,3** | | | 5-mC | |  | | |  | | |  | | | |  | |
| **AA-E10** | | ChmCGG% | | | 0,210 | | | | **21,0** | | | 5-hmC | |  | | |  | | |  | | | |  | |
|  | | CmCGG% | | | 0,326 | | | | **32,6** | | | 5-mC | |  | | |  | | |  | | | |  | |
| **AA-E11** | | ChmCGG% | | | 0,152 | | | | **15,2** | | | 5-hmC | |  | | |  | | |  | | | |  | |
|  | | CmCGG% | | | 0,349 | | | | **34,9** | | | 5-mC | |  | | |  | | |  | | | |  | |
| **AA-12** | | ChmCGG% | | | 0,144 | | | | **14,4** | | | 5-hmC | |  | | |  | | |  | | | |  | |
|  | | CmCGG% | | | 0,292 | | | | **29,2** | | | 5-mC | |  | | |  | | | | | | | |  |
|  |  | | |  | | |  | | | |  | |  | | | | | |  |  |  |  |  |  |  |
|  |  | | |  | | |  | | | |  | |  | | |  | |  | | | |  |  |  |  |

**Supplementary Table 3**

***Pdyn* promoter region methylation (5-mC%) and hydroxymethylation (5-hmC %)**
