# Supplementary material for: Repeated Ethanol Exposure Alters DNA Methylation Status and Dynorphin/Kappa-Opioid Receptor Expression in Nucleus Accumbens of Alcohol-Preferring AA Rats
Source: Front Genet. 2021 Nov 24;12:750142. doi: 10.3389/fgene.2021.750142 (PMC8652212; doi:10.3389/fgene.2021.750142)
Supplement: Supplementary file 4 [file DataSheet2.docx]

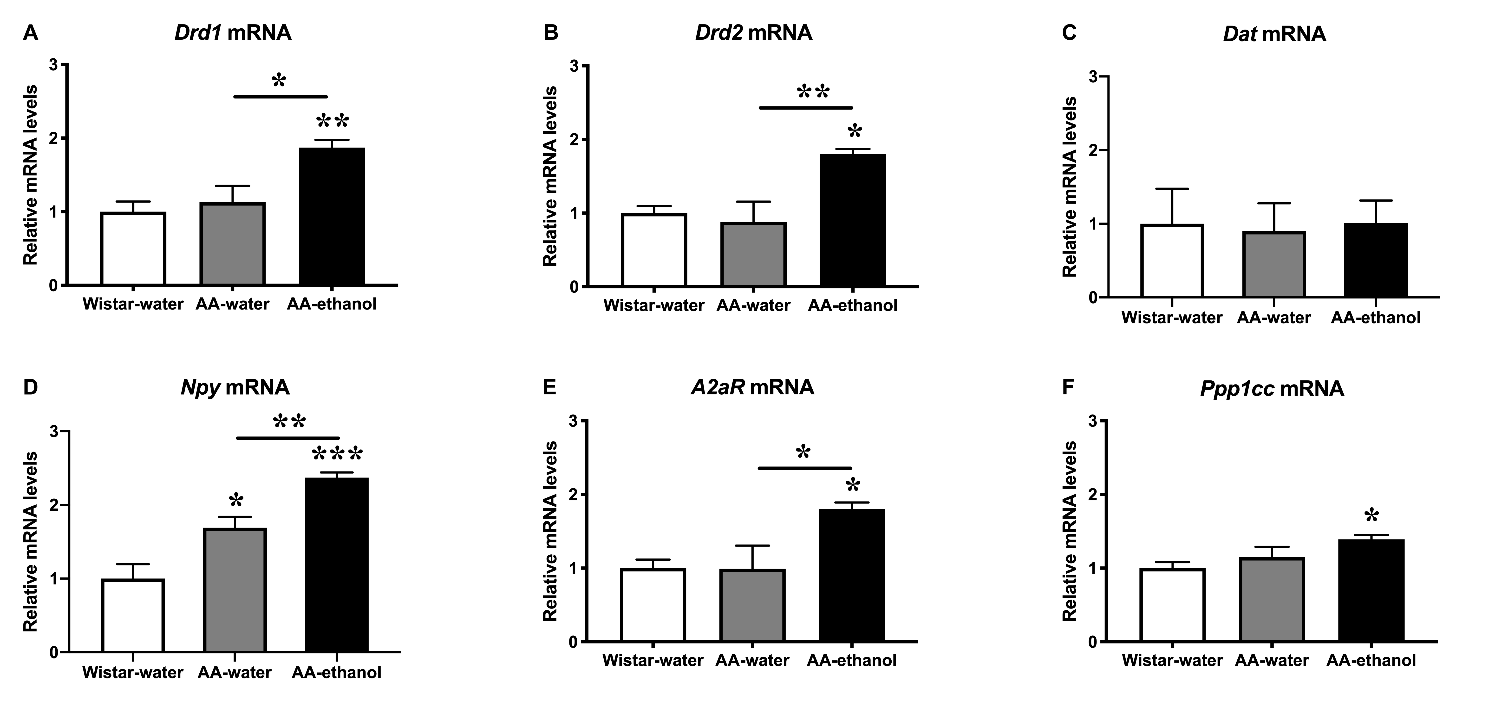


**Supplementary Figure 2.** Impact of intermittent alcohol exposure on *Drd1* (A), *Drd2* (B), *Dat* (C), *Npy* (D), *A2aR* (E), *Ppp1cc* (F) expression levels in the NAc. One-way ANOVA followed by Tukey's multiple comparisons test; ***p<0.001, **p<0.01, *p<0.05 compared with the Wistar-water group; n=5-7. Error bars indicate SEM.
