# Supplementary material for: Repeated Ethanol Exposure Alters DNA Methylation Status and Dynorphin/Kappa-Opioid Receptor Expression in Nucleus Accumbens of Alcohol-Preferring AA Rats
Source: Front Genet. 2021 Nov 24;12:750142. doi: 10.3389/fgene.2021.750142 (PMC8652212; doi:10.3389/fgene.2021.750142)
Supplement: Supplementary file 5 [file Table4.docx]

**Supplementary Table 4**

***Kor* promoter region methylation (5-mC%) and hydroxymethylation (5-hmC %)**

| **Wistar-Water** | |  | |  | | **%** |  | **Group total** | **%** | | stdev | | SEM | |
| --- | --- | --- | --- | --- | --- | --- | --- | --- | --- | --- | --- | --- | --- | --- |
| **W-W36** | | ChmCGG% | | 0,189 | | **18,9** | 5-hmC | **W-W group** |  | |  | |  | |
|  | | CmCGG% | | 0,216 | | **21,6** | 5-mC | 5-hmC | **11,01** | | 5,26 | | 2,35 | |
| **W-W37** | | ChmCGG% | | 0,131 | | **13,1** | 5-hmC | 5-mC | **27,21** | | 3,5 | | 1,55 | |
|  | | CmCGG% | | 0,303 | | **30,3** | 5-mC |  |  |  |  |  |  |  |
| **W-W38** | | ChmCGG% | | 0,066 | | **6,6** | 5-hmC |  |  | |  | |  | |
|  | | CmCGG% | | 0,298 | | **29,8** | 5-mC |  |  | |  | |  | |
| **W-W39** | | ChmCGG% | | 0,061 | | **6,1** | 5-hmC |  |  | |  | |  | |
|  | | CmCGG% | | 0,276 | | **27,6** | 5-mC |  |  | |  | |  | |
| **W-W40** | | ChmCGG% | | 0,103 | | **10,3** | 5-hmC |  |  | |  | |  | |
|  | | CmCGG% | | 0,267 | | **26,7** | 5-mC |  |  | |  | |  | |
|  | |  | |  | |  |  |  |  | |  | |  | |
| **AA-Water** | |  | |  | | **%** |  | **Group total** | **%** | | stdev | | SEM | |
| **AA-W24** | | ChmCGG% | | 0,182 | | **18,2** | 5-hmC | **AA-W group** |  | |  | |  | |
|  | | CmCGG% | | 0,200 | | **20,0** | 5-mC | 5-hmC | **16,35** | | 1,39 | | 0,62 | |
| **AA-W25** | | ChmCGG% | | 0,143 | | **14,3** | 5-hmC | 5-mC | **20,91** | | 3,2 | | 1,42 | |
|  | | CmCGG% | | 0,254 | | **25,4** | 5-mC |  |  |  |  |  |  |  |
| **AA-W26** | | ChmCGG% | | 0,161 | | **16,1** | 5-hmC |  |  |  |  |  |  |  |
|  | | CmCGG% | | 0,222 | | **22,2** | 5-mC |  |  |  |  |  |  |  |
| **AA-W27** | | ChmCGG% | | 0,163 | | **16,3** | 5-hmC |  |  |  |  |  |  |  |
|  | | CmCGG% | | 0,168 | | **16,8** | 5-mC |  |  |  |  |  |  |  |
| **AA-W28** | | ChmCGG% | | 0,169 | | **16,9** | 5-hmC |  |  |  |  |  |  |  |
|  | | CmCGG% | | 0,201 | | **20,1** | 5-mC |  |  | |  | |  | |
|  | |  | |  | |  |  |  |  | |  | |  | |
| **AA-Ethanol** | |  | |  | | **%** |  | **Group total** | **%** | | stdev | | SEM | |
| **AA-E8** | | ChmCGG% | | 0,192 | | **19,2** | 5-hmC | **AA-E group** | |  | |  | |  |
|  | | CmCGG% | | 0,177 | | **17,7** | 5-mC | 5-hmC | **20,30** | | 2,77 | | 1,24 | |
| **AA-E9** | | ChmCGG% | | 0,226 | | **22,6** | 5-hmC | 5-mC | **20,18** | | 6,3 | | 2,81 | |
|  | | CmCGG% | | 0,181 | | **18,1** | 5-mC |  |  | |  | |  | |
| **AA-E10** | | ChmCGG% | | 0,182 | | **18,2** | 5-hmC |  |  | |  | |  | |
|  | | CmCGG% | | 0,167 | | **16,7** | 5-mC |  |  | |  | |  | |
| **AA-E11** | | ChmCGG% | | 0,239 | | **23,9** | 5-hmC |  |  | |  | |  | |
|  | | CmCGG% | | 0,314 | | **31,4** | 5-mC |  |  | |  | |  | |
| **AA-12** | | ChmCGG% | | 0,177 | | **17,7** | 5-hmC |  |  | |  | |  | |
|  | | CmCGG% | | 0,171 | | **17,1** | 5-mC |  |  |  |  |  |  |  |
|  |  | |  | |  |  |  |  |  |  |  |  |  |  |
